# Supplementary material for: KinPred: A unified and sustainable approach for harnessing proteome-level human kinase-substrate predictions
Source: PLoS Comput Biol. 2021 Feb 8;17(2):e1008681. doi: 10.1371/journal.pcbi.1008681 (PMC7895412; doi:10.1371/journal.pcbi.1008681)

Supplemental Figure 1: Within Predictor Similarities

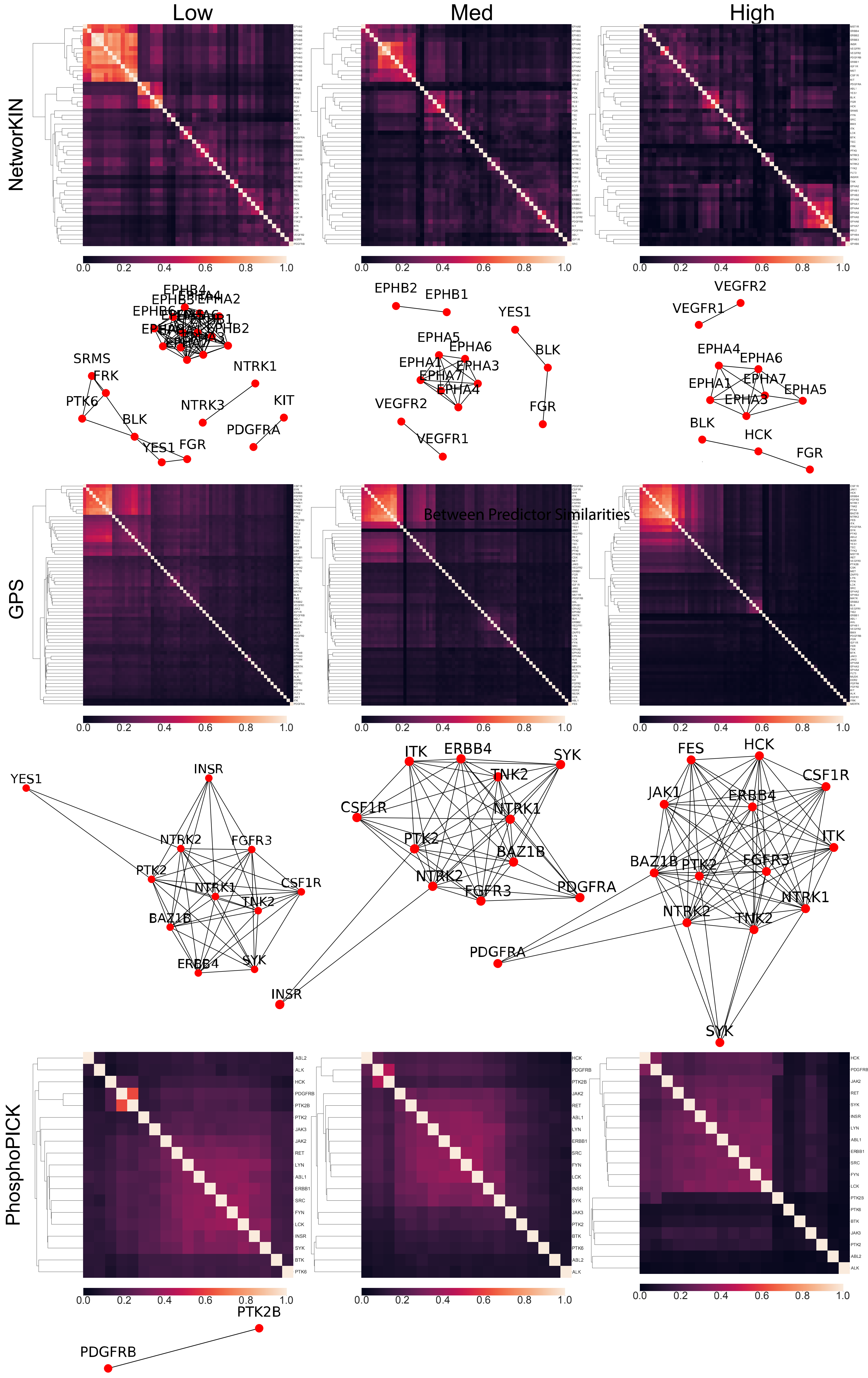

Supplemental Figure 1: Within Predictor Similarities, continued (ser/thr)

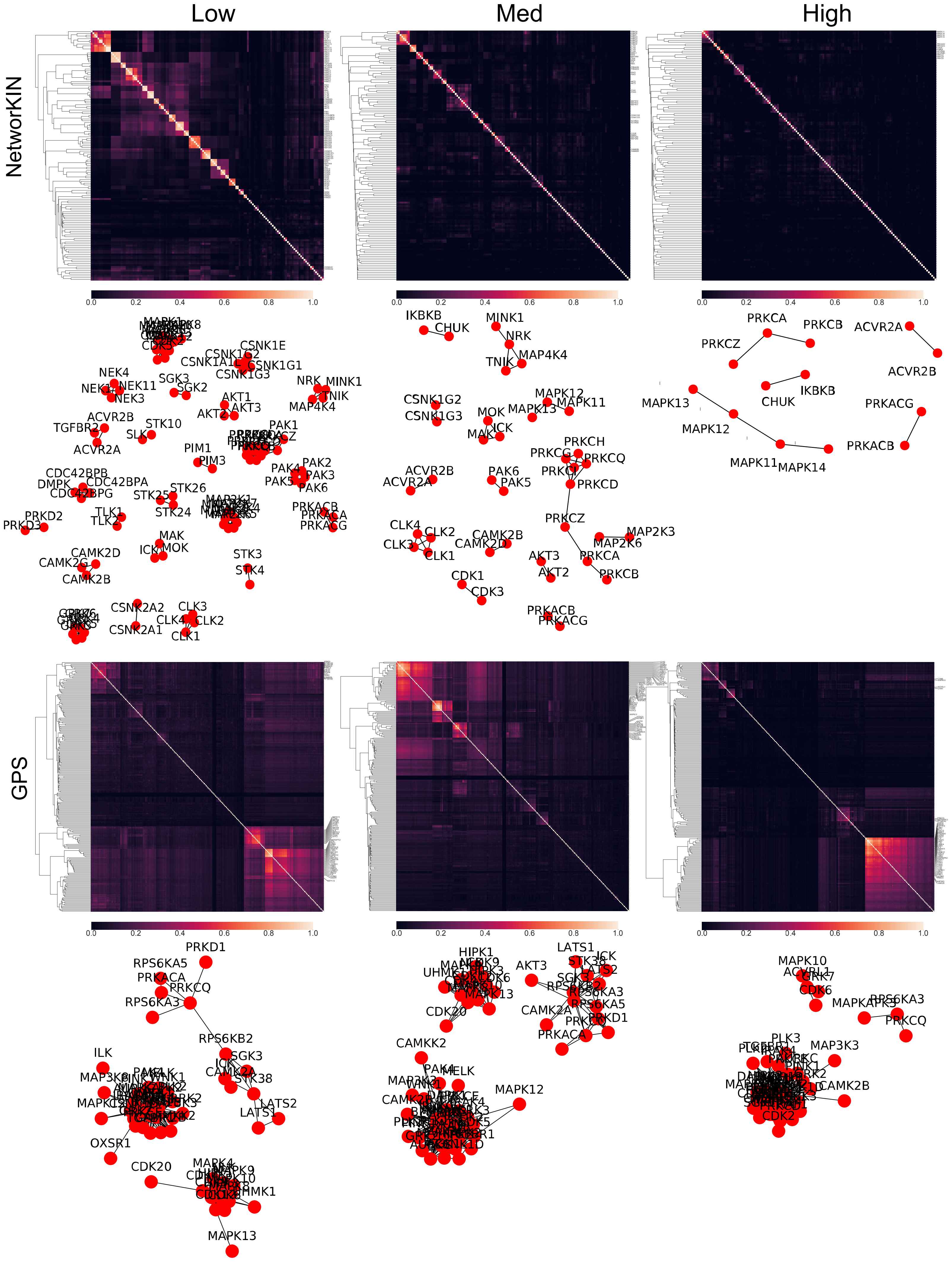

Supplemental Figure 1: Within Predictor Similarities, continued (ser/thr)

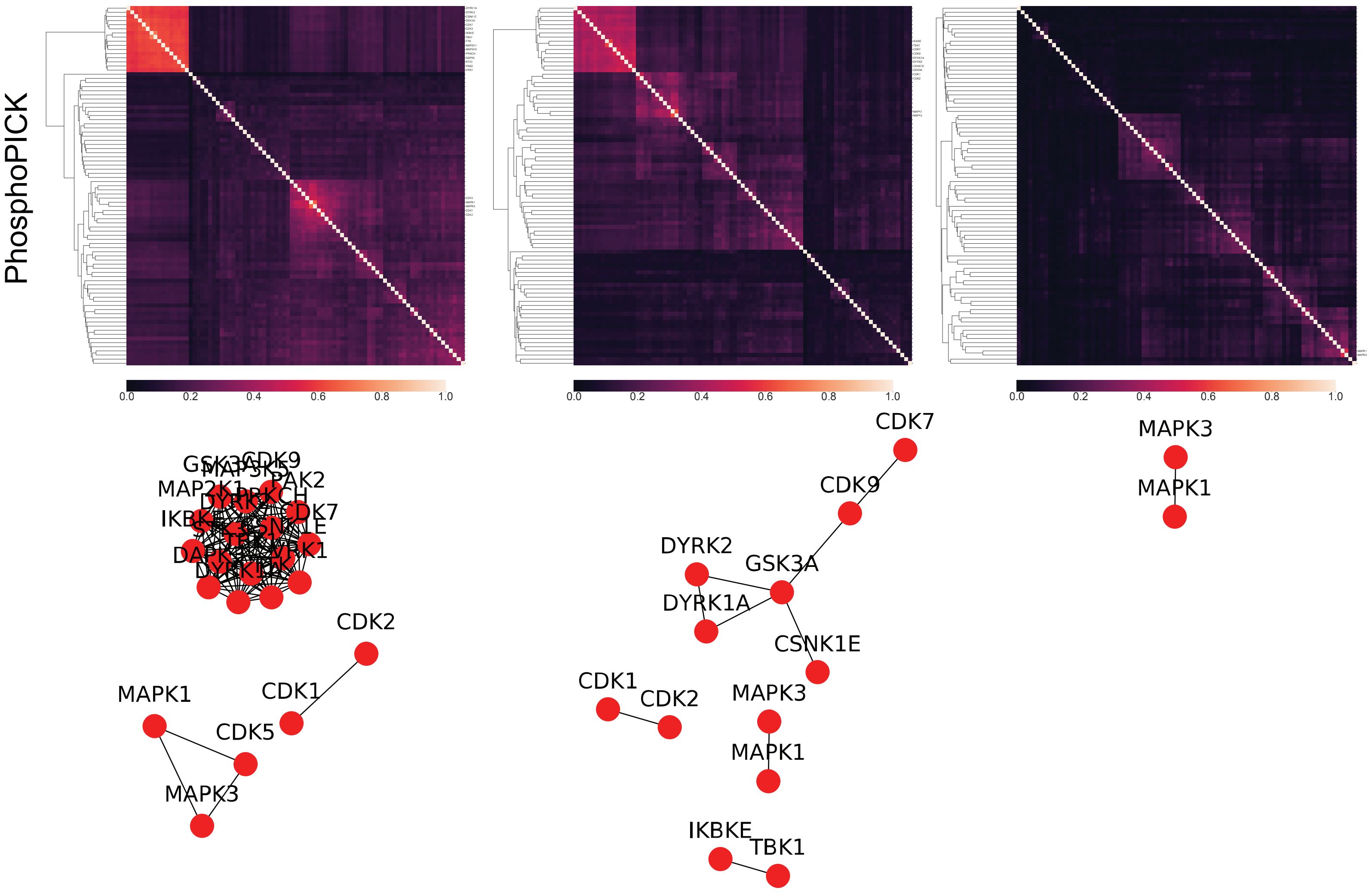

Supplement: S1 Fig — Within-algorithm kinase similarity measured by Jaccard index for all predictive algorithms at all stringencies (low, medium, and high from left to right). The symmetric matrix was sorted hierarchically. The within-algorithm relationships that exceed significant Jaccard index (0.49) values are shown in network diagrams, below their corresponding heatmap. The figures are separated by kinase type (tyrosine kinases first and serine/threonine kinases second. For dense maps, where it is not possible to achieve label separation, the names of kinases can be found as labels in the heatmap, where heatmap sorting correlates with proximity in the network graphs. (PDF) [file pcbi.1008681.s001.pdf]
