# Supplementary figures and images for: KinPred: A unified and sustainable approach for harnessing proteome-level human kinase-substrate predictions
Source: PLoS Comput Biol. 2021 Feb 8;17(2):e1008681. doi: 10.1371/journal.pcbi.1008681 (PMC7895412; doi:10.1371/journal.pcbi.1008681)

Supplemental Figure 2: Between Predictor Comparisons

Tyrosine Kinases

Low

Med

High

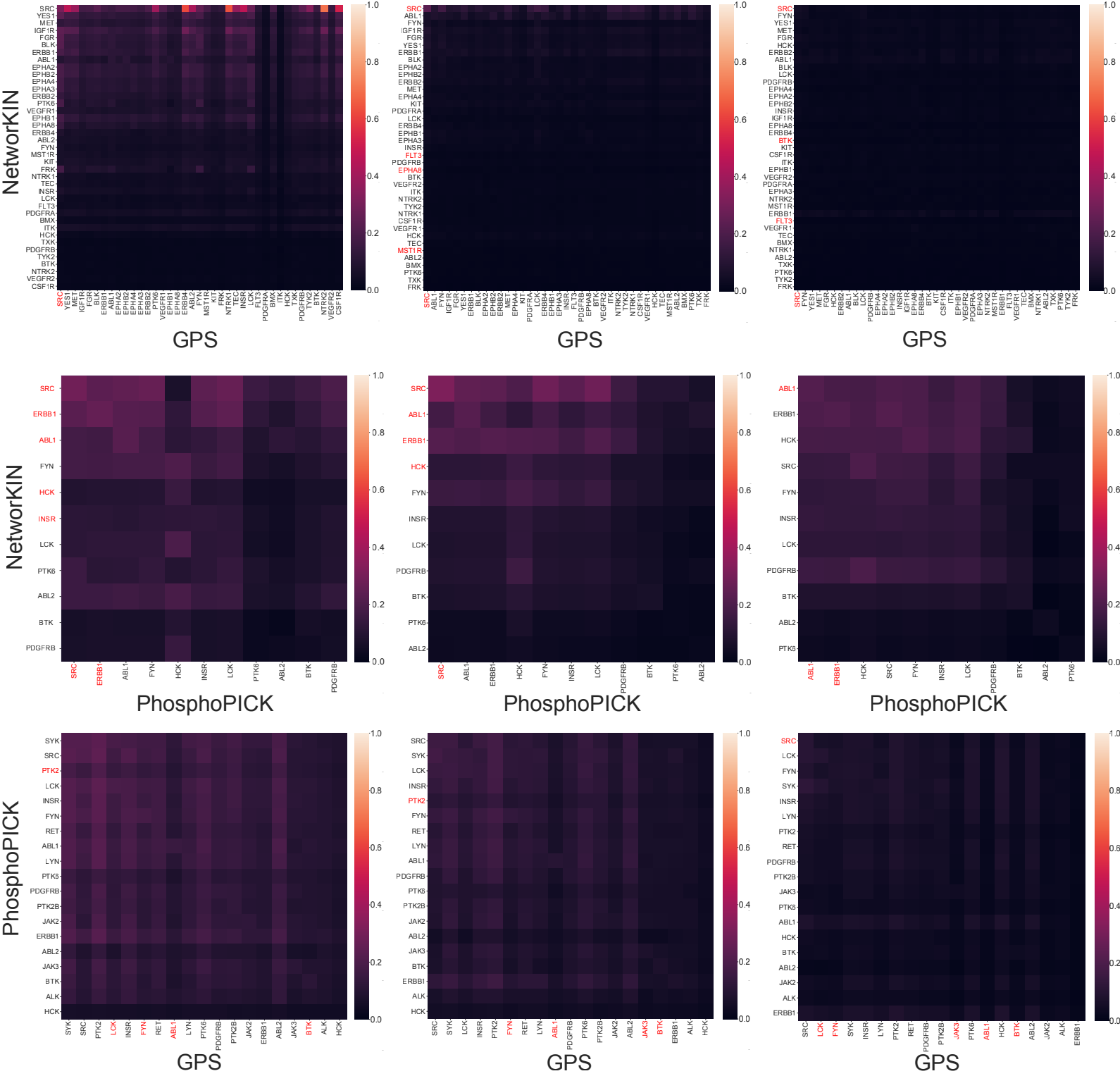

Supplement: S2 Fig — Between-algorithm kinase similarity measured by Jaccard index for all combinations of predictive algorithms at all stringencies (low, medium, and high from left to right). The matrices are sorted by the same kinase order on both the Y- and X-axes. If the ranking of kinase in algorithm A is in the top 1% of similarities in algorithm B comparisons, the font is in red for tyrosine kinases, or indicated with a label in serine/threonine kinases. (PDF) [file pcbi.1008681.s002.pdf]

Supplemental Figure 3: Ranking Performance for Randomized Datasets

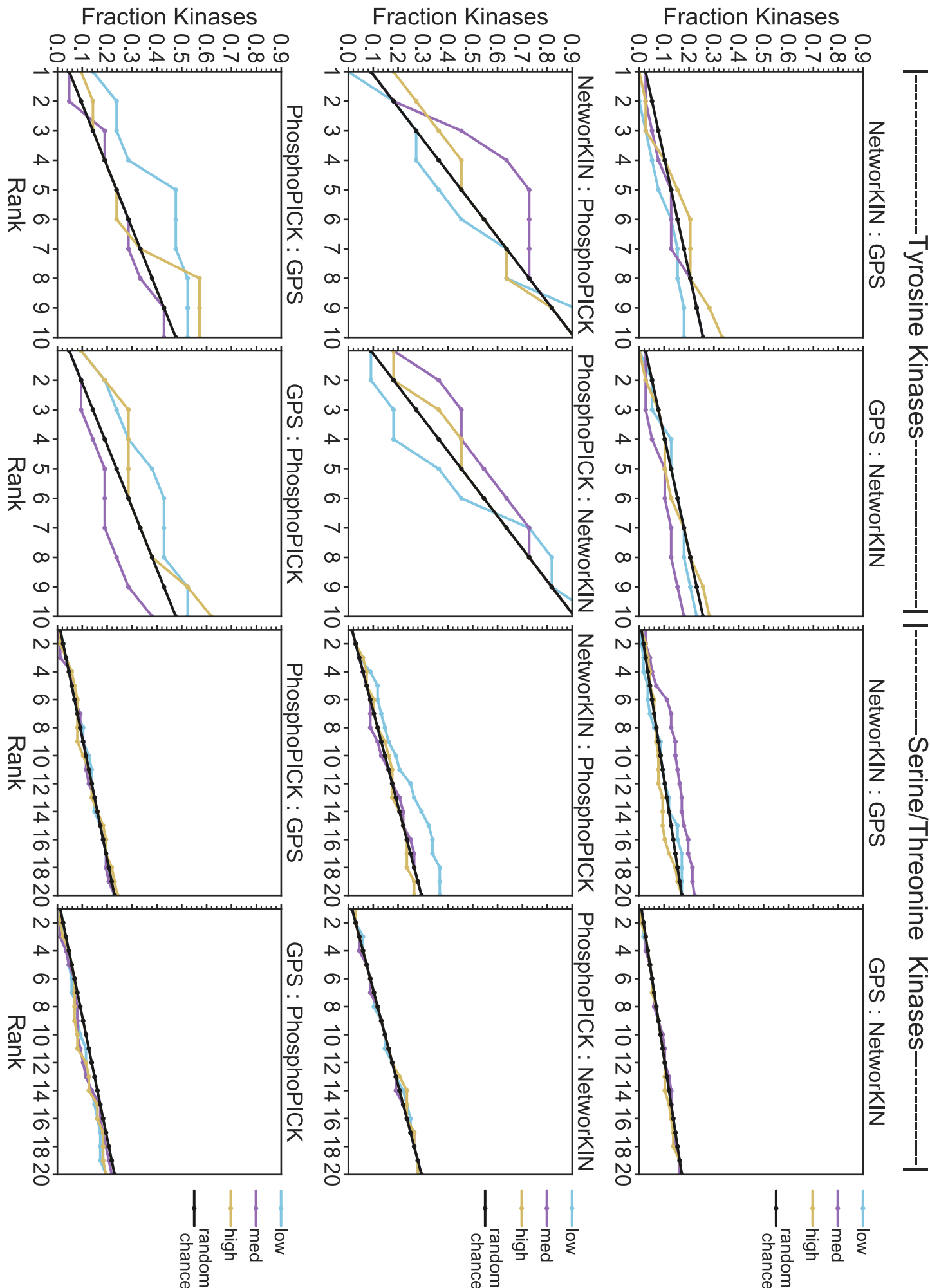

Supplement: S3 Fig — The edges of predictive algorithms were randomized and the process of measuring Jaccard index overlap between predictive algorithms and measuring the cumulative distribution function of rankings, as done in Fig 5 was repeated on random datasets. The dashed-black line indicates the expectation of random performance line. (PDF) [file pcbi.1008681.s003.pdf]
